# A novel coconut-malt extract medium increases growth rate of morels in pure culture

**DOI:** 10.1186/s13568-021-01325-2

**Published:** 2021-12-15

**Authors:** Fabiola Rodríguez Evangelista, Isaac Chairez, Sigfrido Sierra, Hermilo Leal Lara, César Ramiro Martínez-González, María Eugenia Garín Aguilar, Gustavo Valencia del Toro

**Affiliations:** 1 Laboratorio de Cultivos Celulares de la Sección de Estudios de Posgrado e Investigación, UPIBI, Instituto Politécnico Nacional, Barrio la Laguna s/n Ticomán, CP 07340 Mexico City, México; 2 Unidad Profesional Interdisciplinaria de Biotecnología, Instituto Politécnico Nacional, Barrio la Laguna s/n Ticomán, CP 07340 Mexico City, México; 3 Laboratorio de Taxonomía de Hongos Tremeloides (Heterobasidiomycetes), Departamento de Biología Comparada, Facultad de Ciencias, Universidad Nacional Autónoma de México, Ciudad Universitaria, Av. Universidad 3000, Coyoacán, 04510 Mexico City, Mexico; 4 Departamento de Alimentos y Biotecnología, Facultad de Química, Universidad Nacional Autónoma de México, Cd. Universitaria, 04510 Mexico City, México; 5 Posgrado en Biotecnología Agrícola, Instituto de Horticultura, Departamento de Fitotecnia, Universidad Autónoma de Chapingo, Chapingo, 56230 México, Texcoco, Mexico State Mexico; 6 Laboratorio de Farmacobiología, FES Iztacala, Universidad Nacional Autónoma de México, Av. de los Barrios No.1. Los Reyes Iztacala, CP 54090 Tlalnepantla, Mexico State México

**Keywords:** *Morchella*, Coconut water, Cultivation, Baranyi’s model, Growth rate

## Abstract

Morels are gourmet wild edible mushrooms that can grow on several substrates with significant growth rate variations. Such variations have hindered the development of a standardized culture media to promote morel’s sustainable production. The aim of this study is developing a novel culture media that takes advantage of coconut water as a complementary component of culture media. Coconut water has been extensively used as a growth-promoting component for plant tissue cultures; however, its application as component of fungi cultivation medium has not been fully developed. This study confirms that coconut water can be efficiently used as culture media component for morels using a kinetic characterization. *Morchella* sp. kinetic growth is evaluated in different cultures: agar, malt extract agar (MEA), lactose, coconut water (15%) and combinations of them. Kinetic growth parameters (lag phase, λ and maximum specific growth rate, µ_max_) are estimated using primary modeling methods. Among the selected models, the best fit is achieved using Baranyi’s model. A significant increase from 15.8% to 43.4% of the µ_max_ values was observed when culture media (agar, lactose, MEA) is supplemented with coconut water. The largest values of µ_max_ are obtained in MEA-coconut cultures (21.13 ± 0.43–22.57 ± 0.35). Micro-sclerotia and late sclerotia are observed in all cultures containing coconut water justifying the development of a feasible and cost-effective way of culturing morels. The results demonstrate that coconut water can be used for formulation of standard media for morel cultivation leading to a cheap alternative to produce dense mycelium and promote sclerotia formation.

**Supplementary Information:**

The online version contains supplementary material available at 10.1186/s13568-021-01325-2.

## Key points

Coconut water can be used for the formulation of a standard culture media for morels.

Non-linear models are a robust parametric strategy to describe morels growth.

Morel high quality mycelium and sclerotia can be produced by using coconut water.

## Introduction

Morels are edible mushrooms which are highly appreciated worldwide due to their diverse usages in both food and pharmaceutical industries. All species of this genus are edible. Morels are widely distributed in temperate and subtropical forest because they can grow at different temperatures and altitudes (El-Gharabawy et al. 2019; Liu et al. 2017; Loizides 2017; Pilz et al. 2007; Goldway et al. 2000; Guzmán and Tapia 1998). In Mexico, morel fruiting time is from August to October. Due to its culinary and medicinal importance, wild morels are intensively collected implying the overexploitation risk of wild populations (Raut et al. 2019). Moreover, their profitable commerce at local and international markets justifies the necessity of developing artificial cultivation process (Raut et al. 2019; Liu et al. 2017; Du et al. 2015; Pilz et al. 2007).

Commercial artificial cultivation under controlled environmental conditions has not been achieved yet. Nevertheless, outdoors cultivation (including the entailed limitations) is a common practice in China (Liu et al. 2017; Du et al. 2015). The potential drawbacks in outdoor cultivation include low mycelium and sclerotia productivities. One of the main reasons of this significant problem is a consequence of the large variations in growth rates during the first phase of morel cultivation (Guler and Ozkaya 2008). The variations of growing capabilities are associated to fungal species, isolates and culture media (Guler and Ozkaya 2008; Winder 2006; Hervey et al. 1978). Despite morel mycelium grows on a wide variety of substrates (Brock 1951), there is no consensus regarding a standard culture broth formulation for morel in vitro mycelial growth.

Since the presence of sclerotia is an essential condition for the fruiting bodies formation, their production is a relevant step during morel cultivation (Liu et al. 2018; Kanwal and Reddy 2012; Guler and Ozkaya 2008; Winder 2006; Amir et al. 1992, 1993, 1995; Buscot 1993; Volk and Leonard 1989; Ower et al. 1986; Ower 1982). Sclerotia are defined as resistance structures against hostile environmental conditions (Pilz et al. 2007; Georgiou et al. 2006). Usually, they appear in *Ascomycetes* and *Basidiomycetes* (Georgiou et al. 2006). True sclerotia are nodules of differentiated mycelium, but in morels these structures are pseudosclerotia or false sclerotia because the mycelium is only a mass of undifferentiated hyphae (Pilz et al. 2007; Volk and Leonard 1990). Although the most appropriate term would be “pseudosclerotia”, the term “sclerotia” on morels was adopted for simplicity (Pilz et al. 2007). Due to the importance of sclerotia, nearly all studies have been focused on promoting sclerotia formation (Liu et al. 2018; Kanwal and Reddy 2012; Guler and Ozkaya 2008; Winder 2006; Amir et al. 1992, 1993, 1995; Buscot 1993) leaving aside the production of good quality mycelium and description of mycelial growth. Furthermore, development of a standard culture media for morel has not been properly tackled.

Numerous culture media have been tested with the aim of promoting large amounts of sclerotia. Several variations on carbon and nitrogen sources were tested, but sclerotia production was neither constant nor related enough to the carbon/nitrogen ratio (Kanwal and Reddy 2012; Guler and Ozkaya 2008; Buscot 1993). When a considerable amount of sclerotia was produced, the culture media were either very expensive or difficult to formulate. Thus, formulation of cost-effective culture media for high scale cultivation process has not been achieved yet.

Among the supplementary compounds added to culture media, coconut water has been extensively used as a growth-promoting component for plant tissue cultivation (Winarto and da Silva 2015; Prando et al. 2014; Peixe et al. 2007). Despite coconut water increased growth rate of few species of *Basidiomycetes* (Zurbano et al. 2017; Jacob et al. 2015; Magday et al. 2014) and increased biomass production of Ascomycete *Ophiocordyceps sinensis* (Shashidhar et al. 2017), its use as a culture supplement for mushroom cultivation has not been fully developed.

In this study:

- it is demonstrated that utilization of coconut water as a supplement culture media enhances growth rate of morel mycelium.
- sclerotia production can be achieved by supplementing the culture media with coconut water.
- morel mycelium growth by using primary modeling is described for the first time.

## Methods

### Fungal isolates

Morel mycelia were isolated from six wild ascomata collected during September- October 2018 and August 2019 at locations listed in Table 1. Two ascomata of *Morchella* sp. were bought on September 2018 at the local market Melchor Múzquiz in Mexico City. According to the information provided by the seller, the ascomata came from Río Frio, Puebla. Ascomata from San Pedro Tlalcuapan were donated at the Vth Festival for the Culture of Wild Mushrooms, held from August 29 to September 1, 2019. Description data of ascomata, strains and molecular data are in process of publication.

**Table 1:** Morel ascomata collected from different regions of central Mexico

Ascoma and isolate ID	Ascoma	Location	Habitat	Date of collection
FRE-3C *Morchella* sp.		National Park Izta-Popo Zoquiapan, Estado de México (19º17′27"N, 98º40′2"W)	Coniferous forest	22/09/2018
FRE-5E *Morchella* sp.		Local market Melchor Múzquiz, Mexico City (near Río Frío, Puebla. 19º12′22"N, 99º14′36"W)*	–	24/09/2018
FRE-6F *Morchella* sp.		Local market Melchor Múzquiz, Mexico City (near Río Frío, Puebla. 19º12′22"N, 99º14′36"W)*	–	24/09/2018
FRE-I *Morchella* sp.		Ajusco Summits National Park (19º12′22"N, 99º14′36"W)	Coniferous forest	24/10/2018
FRE-L *Morchella* sp.		Near San Pedro Tlalcuapan, Tlaxcala (19º16′45"N, 98º8′47"W)**	Coniferous-oak forest	30/08/2019
FRE-M *Morchella* sp.		Near San Pedro Tlalcuapan, Tlaxcala (19º16′45"N, 98º8′47"W)**	Coniferous-oak forest	30/08/2019

^*^Ascomata were bought in the local market Melchor Muzquiz commonly known as Mercado de San Ángel in Mexico City. According to the information provided by the seller the ascomata came from near Río Frio, Puebla

^**^Ascomata were donated at the V Festival for the Culture of Wild Mushrooms

*Morchella crassipes* CDBB-H-482 reference strain (World Data Centre for Microorganisms of World Federation for Culture Collections) also referred as *Morchella esculenta,* was acquired at the National Collection of Microbial Cell Strains of the Advanced Studies Research Center of the Instituto Politécnico Nacional and incorporated to all the experiments for comparison purposes. In the present study CDBB-H-482 strain was referred as *M. esculenta* instead of *M. crassipes*.

### Isolating procedures

Collected ascomata were dried and stored at 4 °C until analysis. Ascospores of each dried ascoma were obtained by cutting a slice of the ridge under standard conditions. Dry tissue was transferred into a sterilized tube containing an antibiotic solution (neomycin, 1.75 mg mL^−1^; polymyxin B, 5000 UI; bacitracin, 0.025 mg mL^−1^) and incubated for 30 min. The tissue was transferred into another tube containing sterile distilled water. The tissue was shacked up several times and a set of dilutions were prepared to obtain 100–300 ascospores per 1000 µL of sterile distilled water. An aliquot of 100 µL of the ascospore suspension and an aliquot of 100 µL of the antibiotic solution were placed in Petri dishes. The culture media contained 12 gL^−1^ of bacteriological agar and coconut water (15%). After four of five days, each culture was sub-cultured on malt extract agar (MEA) and stored for growth tests. Coconut water-agar media (containing 12 gL^−1^, 15%) was chosen because attempts to germinate ascospores in potato dextrose agar (PDA) and MEA were most of times unsuccessful. In all the experiments, coconut water was obtained from immature nuts of approximately six months old (Prades et al. 2012) containing very few flesh and large amounts of water.

### Experimental design

To assess growth rate, the experiment was divided into two stages. At the first stage, seven different culture media were tested using one wild strain (isolate FRE*-*3C) and the reference strain (*M. esculenta,* CDBB-H-482). For the second stage, the optimal culture medium was chosen to grow all wild morel isolates: FRE-5E, FRE-6F, FRE-I, FRE-L and FRE-M. Ten replicates were performed per treatment.

### Culture media

The fundamental culture media were prepared using agar medium (20 gL^−1^), agar-coconut water, hereafter referred to as “coconut” (20 gL^−1^, 15%), agar malt extract (20 gL^−1^, 20 gL^−1^) and agar-lactose (20 gL^−1^, 0.037 M). Lactose was incorporated to the culture broths due to it is considered a low-cost but efficient carbon source for morel cultures (Winder 2006).

Combinations of agar-lactose-conut, MEA-coconut and agar-MEA-lactose were also prepared. All culture media were autoclaved at 121 °C (15 psi) for 20 min. A volume of 15 mL of sterile medium was poured into Petri dishes (90 × 15 mm) and incubated at 28 °C for 48 h for sterility test. Mycelium circular inocula (~ 0.8 cm diameter) of morel strains were transferred from a fresh MEA growing culture to Petri dishes containing the different culture broths. All the cultures were incubated at 20–25 °C in darkness. Growth was calculated by measuring the radial growth of the mycelium every day for a period of 3 to 6 days using a non-linear parameterized modeling strategy based on different fungal growth models.

### Morel mycelial growth kinetics

With the aim of having a better understanding of morel mycelium growth, all the experiments carried out were fitted by using primary modeling. Linear, Baranyi’s and modified Logistic models (Table 2) were tested in order to use the traditional models of fungal growth. The selection of these three models allows description of growth curves with different levels of accuracy of the corresponding kinetic parameters (Marín et al. 2008; Baty and Delignette-Muller 2004; Zwietering et al. 1990). The inner comparison of such kinetic parameters obtained for each model yields to characterize the effect of introducing coconut water as an additional component of the culture broth for morels.

**Table 2:** Selected mathematical model for characterizing kinetic growth

Mathematical model	Mathematical expression
Linear model	$y\left( t \right) = \mu_{max} t + y_{max}$
Baranyi’s model	$y\left( t \right) = y_{max} + {\text{ln}}\left( {\frac{{ - 1 + e^{{\mu_{max} \lambda }} + e^{{\mu_{max } t}} }}{{\left( { - 1 + e^{{\mu_{max} t}} } \right) + e^{{\left( {\mu_{max} \lambda + y_{max} - y_{0} } \right)}} }}} \right)$
Logistic modified model	$y\left( {t_{max} } \right) = \frac{A}{{1 + e^{{\left[ {\frac{{4\mu_{max} }}{A}\left( {\lambda - T} \right) + 2} \right]}} }}$

In the models included in Table 2, $\mu_{max}$ = maximum growth rate (mm d^−1^), $y_{max}$ = maximum diameter attained, $y_{0}$ = initial diameter or inoculum diameter, $t$ = time, $\lambda$ = lag phase before visible growth (d), e = 2.7183 and $A$ = is the upper asymptotic value.

## Modeling strategy

Considering that the selected models are defined with nonlinear parameters (including the linear form), an online adaptive parametric estimation method was proposed based on the Levenberg–Marquardt method.

The method used the following procedure:

1. Collect data $y\left( {kT} \right)$ with $k$ defining the sampled radius of the morel growth in the Petri dish, and $T$ the sampling period (one day).
2. Implement an interpolation method based on splines approximation over a time partition $k_{i} , i = 1:N_{i}$ based on $y\left( {kT} \right)$ that generates a new vector $y_{i} \left( {k_{i} T_{i} } \right)$ with $T_{i}$ the new interpolating period.
3. Using the new information $y_{i} \left( {k_{i} T_{i} } \right)$, define the performance index for the modeling strategy defined as
  $$J = \sum\limits_{i = 1}^{{N_{i} }} {\left( {y\left( {k_{i} T_{i} } \right) - F_{i} \left( {\widehat{\mu }_{\max } ,\widehat{\lambda }} \right)} \right)^{2} }$$
  where $F\left( {\mu_{max} ,\lambda } \right)$ is any of the selected models in Table 2 evaluated at the movement $k_{i}$.
4. Based on the Matlab regression toolbox, the application of the Levenberg–Marquardt yields to define the evolution of the estimated parameters $\left( {\hat{\mu }_{max} ,\hat{\lambda }} \right)$ in the model which are defined as
  $$\begin{array}{*{20}c} {\hat{\mu }_{{max,\left( {i + 1} \right)}} = \hat{\mu }_{max,\left( i \right)} + \left[ {\mu I + S_{{\hat{\mu }_{max} }}^{T} S_{{\hat{\mu }_{max} }} } \right]^{ - 1} S_{{\hat{\mu }_{max} }}^{T} \left( {\mathop{y}\limits^{\rightharpoonup} - \mathop{F}\limits^{\rightharpoonup} \left( {\hat{\mu }_{max} ,\hat{\lambda }} \right)} \right)} \\ {\hat{\lambda }_{{\left( {i + 1} \right)}} = \hat{\lambda }_{\left( i \right)} + \left[ {\mu I + S_{{\hat{\lambda }}}^{T} S_{{\hat{\lambda }}} } \right]^{ - 1} S_{{\hat{\lambda }}}^{T} \left( {\mathop{y}\limits^{\rightharpoonup} - \mathop{F}\limits^{\rightharpoonup} \left( {\hat{\mu }_{max} ,\hat{\lambda }} \right)} \right)} \\ \end{array}$$
  where $\hat{\mu }_{{max,\left( {i + 1} \right)}}$ and $\hat{\lambda }_{{\left( {i + 1} \right)}}$ are the estimated values at the upcoming moment of the parameters of interest, $\mu$ is a selected positive scalar, $\mathop{y}\limits^{\rightharpoonup}$ is the vector made of the measurements $y_{i} \left( {k_{i} T_{i} } \right)$ while $F_{i} \left( {\hat{\mu }_{max} ,\hat{\lambda }} \right)$ is the corresponding vector made with the values of $F_{i} \left( {\hat{\mu }_{max} ,\hat{\lambda }} \right)$. The functions $S_{{\hat{\mu }_{max} }}$ and $S_{{\hat{\lambda }}}$ are the derivatives of $J$ with respect to $\hat{\mu }_{max}$ and $\hat{\lambda }$.
5. The evolution of the algorithms presented in the step 4 allows to estimate the parameters under study, that is
  $$\hat{\mu }_{max}^{*} = \hat{\mu }_{{max,\left( {N_{i} + 1} \right)}}, \; \text{and} \; \hat{\lambda }^{*} = \hat{\lambda }_{{\left( {N_{i} + 1} \right)}},$$

For the election of the primary model that best described growth, the squared errors of each model were compared. The model that showed the lowest mean quadratic errors (Ross 1996) was chosen for further statistical analysis. Notice that this strategy allowed not only comparing the obtained modeling results in this study, but compare them with respect to other modeling effort of morel growth experiments.

### Statistical analysis

Growth differences among different culture media, strains and models including maximum growth rate (μ_max_) and lag phase (λ) were compared using the multivariate analysis of variance (MANOVA). When statistical differences were found, the Duncan Test with α = 0.05 was applied.

## Results

### MEA-coconut media resulted in the highest mycelial growth

With the purpose of selecting a culture broth that promotes the highest growth rate (among the evaluated media) of morel mycelium, the reference strain *M. esculenta* (for reference purposes) and the wild isolated FRE-3C (Table 1) were cultivated in seven different culture media (agar, agar-lactose, agar-coconut, agar-lactose-coconut, MEA, MEA-lactose and MEA-coconut).

**Fig. 1:**
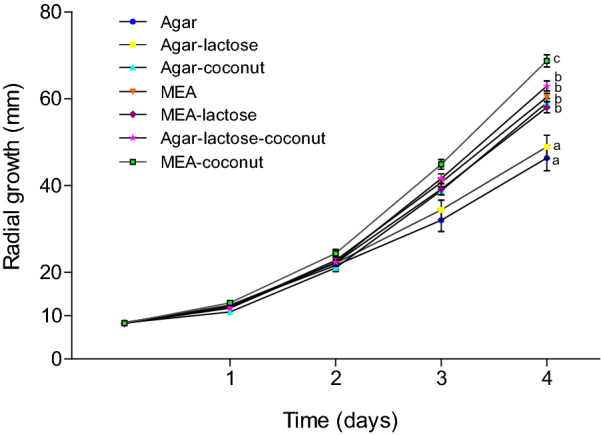
Mean radial growth (mm) of *M. esculenta* (CDBB-H-482) and FRE-3C in different culture media (n = 20)

Morel growth was estimated using the maximum radius of mycelium distribution in the Petri dish, measured every day. Qualitative characteristics as color, mycelial density and presence of aerial or submerged mycelium were also described. No effect of strain type (*M. esculenta* and wild morel isolate FRE-3C*)* on growth was observed (*p* < 0.05); therefore both strains were considered as homogeneous group to assess the effect of culture media on mycelial growth. Morel strains (*M. esculenta* and wild morel isolate FRE-3C) cultivated in MEA-coconut showed the highest growth (Fig. 1). The lowest growth was registered in agar and in agar-lactose cultures (*p* < 0.05). As MEA-coconut media resulted in the best mycelial growth (Fig. 1), this media was chosen to cultivate all other wild strains (isolates FRE-5E, FRE-6F, FRE-I, FRE-L and FRE-M) for the second part of the growth experiments.

**Fig. 2:**
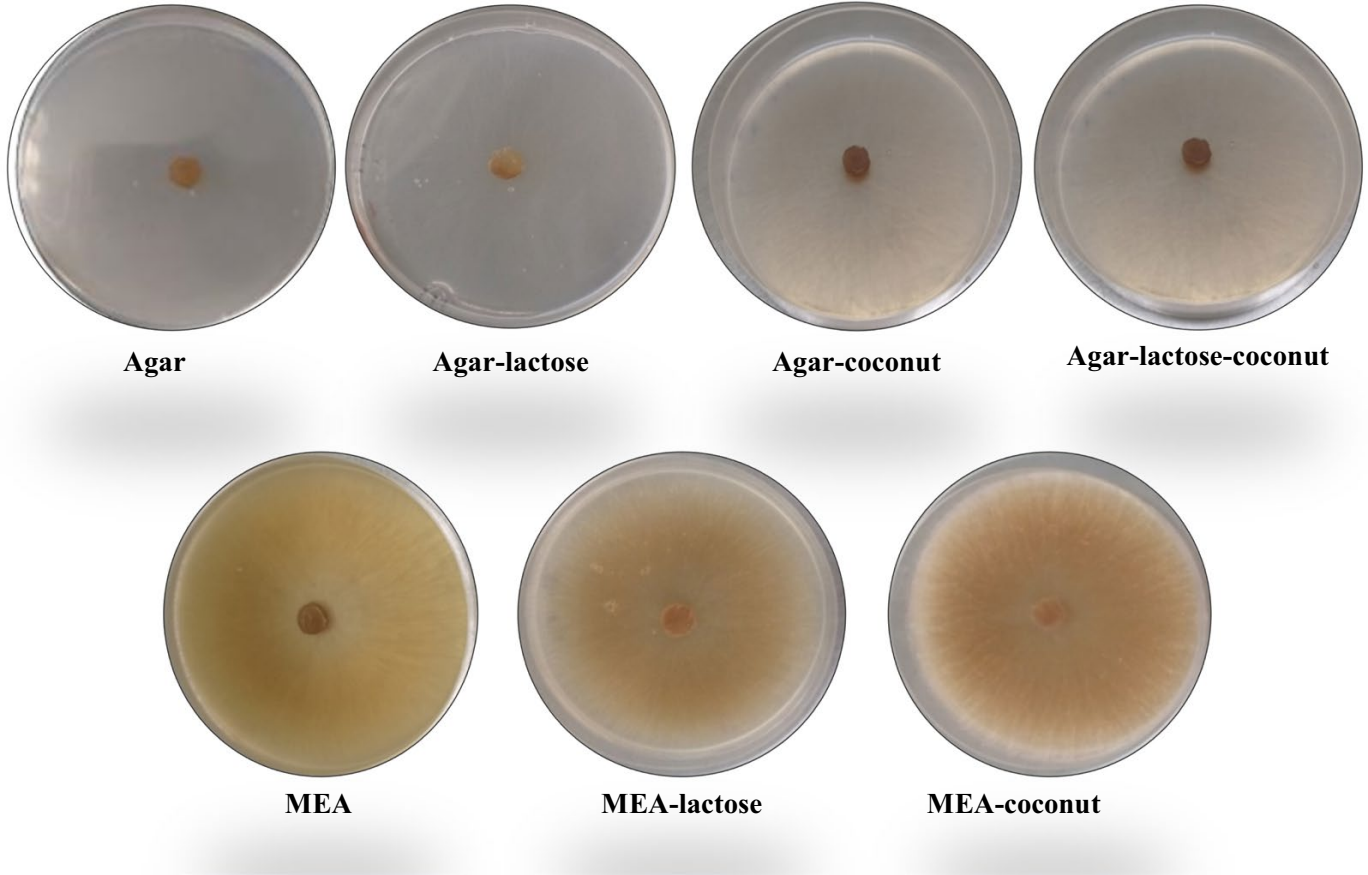
Morphology of *M.esculenta* (CDBB-H-482) cultured in 90 mm Petri dishes on different culture media. All cultures were photographed ten days after inoculation

**Fig. 3:**
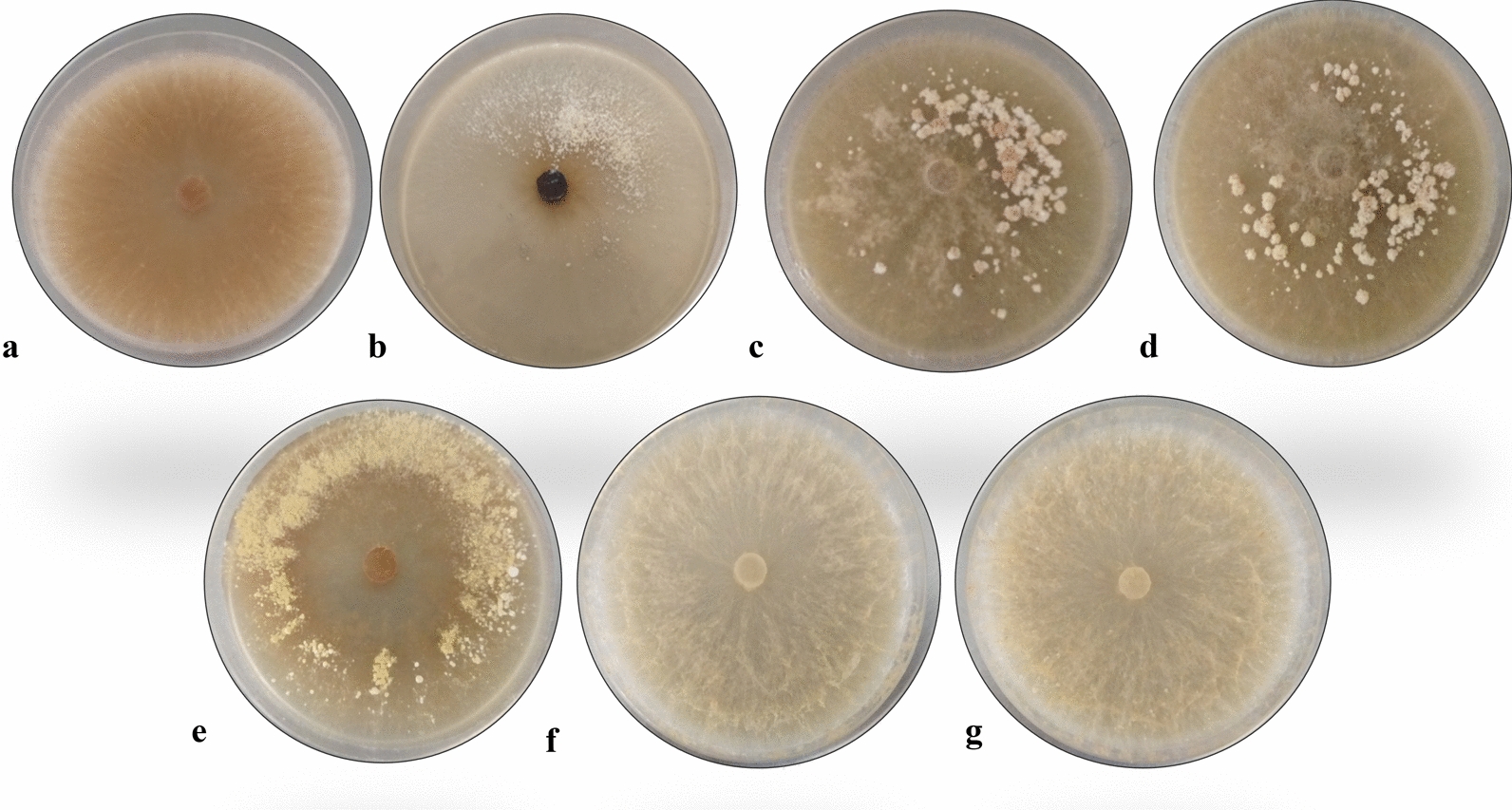
Reference strain and wild morel isolates cultured in 90 mm Petri dishes of MEA-coconut. All cultures were photographed fifteen days after inoculation. **a** *M. esculenta* CDBB-H-482, **b** isolate FRE-3C, **c** isolate FRE-5E*,* **d** isolate FRE-6F, **e** isolate FRE-I, **f** isolate FRE-L, **g** isolate FRE-M

Morphology of morel mycelia changed depending on the culture media. Cultures of agar and agar-lactose grew disperse and hyphae were hyaline. On the other hand, cultures grown in MEA, MEA-lactose and MEA-coconut showed slight brown or brown pigmentations. It should be noticed that sometimes strains showed a slight brown pigmentation and over time turned into brown or dark brown (Table 3, Fig. 2). The largest mycelial density and aerial mycelium were registered in MEA-coconut cultures (Table 3, Figs. 2, 3).

**Table 3:** Morphology description of various morel strains cultivated in different culture media

Culture media	Strain	Color	Mycelium density	Presence of	
				Aerial mycelium	Sclerotia
Agar	*M. esculenta* CDBB-H-482	Hyaline	Very low	0	0
	Isolate FRE-3C	Hyaline	Very low	0	0
Agar-lactose	*M. esculenta* CDBB-H-482	Hyaline	Very low	0	0
	Isolate FRE-3C	Hyaline	Very low	0	0
Agar-coconut	*M. esculenta* CDBB-H-482	Very light brown	Low	0	0
	Isolate FRE-3C	Very light brown	Low	0	1
Agar-lactose-cocount	*M. esculenta* CDBB-H-482	Very light brown	Low	0	0
	Isolate FRE-3C	Very light brown	Low	0	1
MEA	*M. esculenta* CDBB-H-482	Light brown*	Regular	0	0
	Isolate FRE-3C	Very light brown**	Regular	0	0
MEA-lactose	*M. esculenta* CDBB-H-482	Very light brown*	Regular	0	0
	Isolate FRE-3C	Very light brown**	Regular	0	0
MEA-coconut	*M. esculenta* CDBB-H-482	Light brown	Abundant	2	0^†^
	Isolate FRE-3C	Slightly brown*	Abundant	1	1
	Isolate FRE-5E	Light brown	Abundant	3	1
	Isolate FRE-6F	Light brown	Abundant	3	1
	Isolate FRE-I	Light brown**	Abundant	1	1
	Isolate FRE-L	Very light brown	Very abundant	4	1
	Isolate FRE-M	Very light brown	Very abundant	4	1

^†^Presence of young sclerotia (Volk and Leonard 1990)

^*^Over time mycelium turns into brown

^**^Over time mycelium turns into dark brown

Morphological differences among the rest of the isolates were also analyzed. Wild isolates FRE-5E, FRE-6F, FRE-L and FRE-M showed abundant plumose aerial mycelium and slight brown pigmentation whereas *M. esculenta* as well as isolates FRE-3C and FRE-I showed the smaller amounts of aerial mycelium (Table 3, Fig. 3).

All cultures containing coconut water showed the presence of sclerotia after nine to twelve days of cultivation, excepting *M. esculenta* cultures which showed only young sclerotia (Volk and Leonard 1990) on MEA-coconut. Isolates FRE-L and FRE-M exhibited presence of micro-sclerotia (< 0.5 mm; Buscot 1993) at the borders of the Petri dish. Isolates FRE-3C and FRE-I exhibited micro-sclerotia around the center and in some cases in a certain area of the Petri dish. Late sclerotia (> 0.5 mm; Buscot 1993) appeared in cultures of isolates FRE-5E and FRE-6F (Table 3, Fig. 3).

This study shows for the first time the presence of sclerotia in all wild morel isolates cultures containing coconut water, excepting *M. esculenta* cultures in which only young sclerotia (Volk and Leonard 1990) were observed on MEA-coconut.

### Baranyi’s primary model fit the best description of morels mycelial growth

With the aim of characterizing the morel mycelium growth curve, linear, Baranyi’s and exponential models were considered in this study. By using these mathematical models, it was possible to estimate kinetic parameters including lag phase (λ) and maximum specific growth rate (µ_max_) of each cultured strain/isolate. All values of μ_max_ and λ for each model are listed in Table S1 of  Additional file 1. The lowest values of λ were obtained by the linear model while the largest ones were obtained by logistic and Baranyi’s models (*p* < 0.05). The largest values of μ_max_ were obtained using the logistic model and the lowest values were obtained by the linear model (*p* < 0.05). Moreover, logistic model tends to overestimate μ_max_ and, in some cases the predicted values of A (upper asymptotic value) were above of what is biologically possible to expand for a fungi at growing phase. Despite logistic model gave accurate estimations of final diameter of morel colonies and showed a satisfactory fitting quality, Baranyi’s model was chosen for further analysis because it showed the lowest mean quadratic errors (Ross 1996).

### Morel kinetic growth parameters are influenced by culture media

**Fig. 4:**
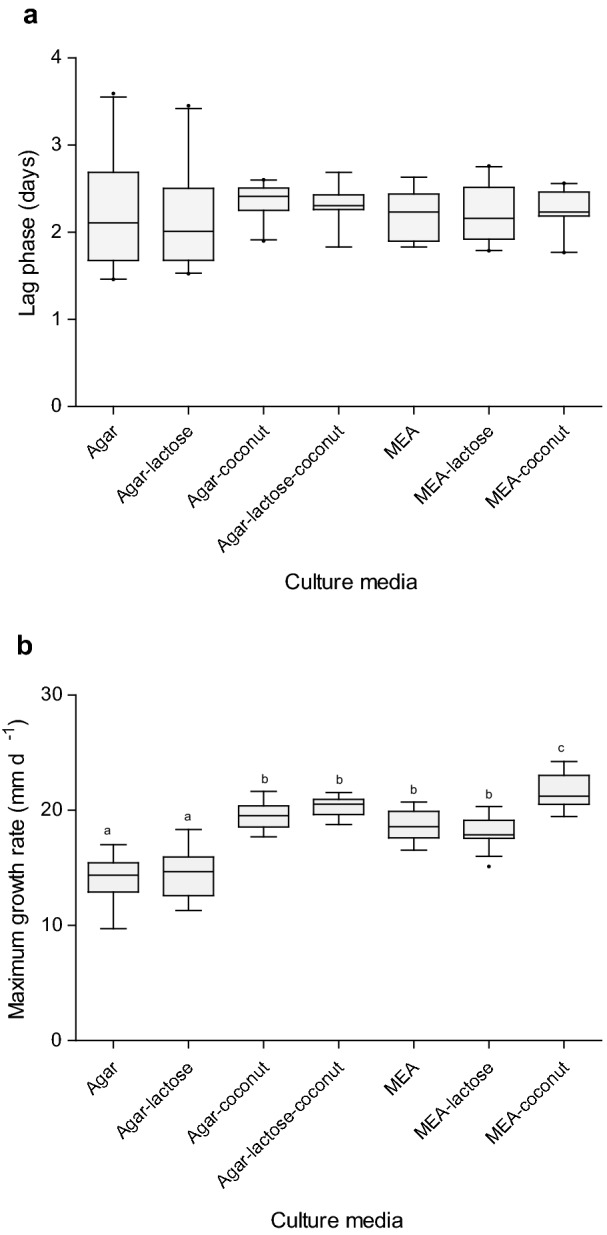
**a** Lag phase of *M. esculenta* CDBB-H-482 and isolate FRE-3C according to culture media. **b** Growth rates of *M. esculenta* CDBB-H-482 and isolate FRE-3C according to culture media. Different letters indicate significant differences according to Duncan test (*p* < 0.05, n = 10)

To elucidate how morel mycelium growth was influenced by culture media, kinetic parameters obtained by Baranyi’s model (μ_max_ and λ) of *M. esculenta* and isolate FRE-3C cultivated in different culture media, were compared by multivariate analysis of variance (MANOVA) and Duncan pos hoc analysis (α = 0.05). Values of lag phase (λ) for both strains did not show differences related to culture media (Fig. 4a, p < 0.05). On the other hand, maximum growth rate was influenced by the selected culture broth. The lowest values of μ_max_ were obtained in agar and agar-lactose cultures and highest ones were obtained in MEA-coconut cultures. In addition, cultures such as agar, agar lactose and EMA increased the values of μ_max_ under presence of coconut water (Fig. 4b, p < 0.05).

### Kinetic growth parameters vary according to the morel species

**Fig. 5:**
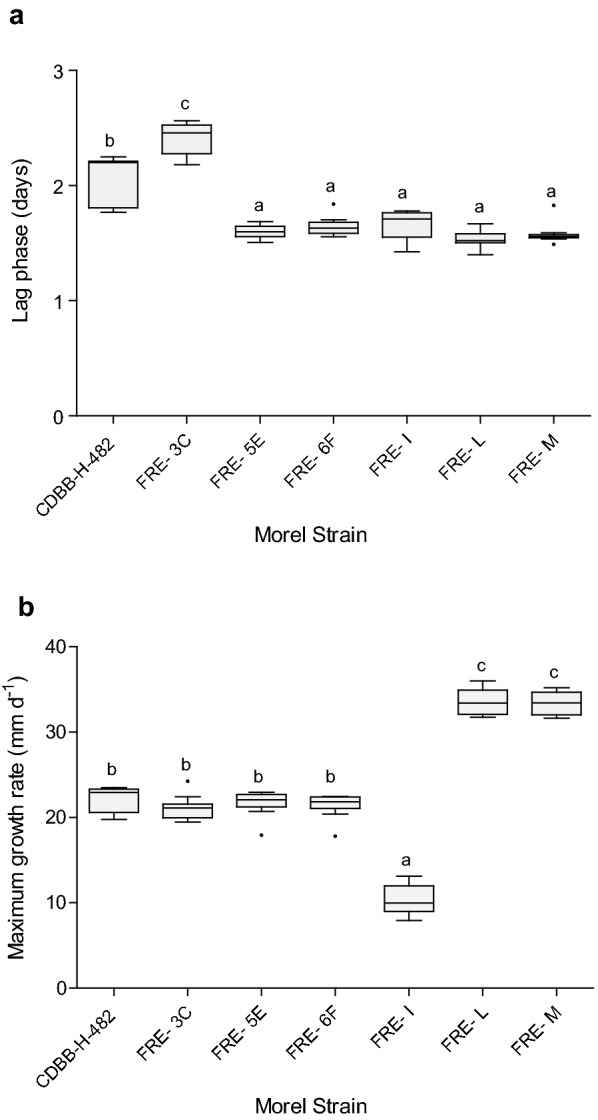
**a** Lag phase of *M. esculenta* CDBB-H-482 and *Morchella* sp. wild isolates (FRE-3C, FRE-5E, FRE-6F, FRE-I, FRE-L, FRE-M) cultivated in MEA-coconut. Different letters indicate significant differences according to Duncan test (*p* < 0.05, n = 10). **b** Growth rates of *Morchella* spp. wild strains cultivated in MEA-coconut. Different letters indicate significant differences according to Duncan test (*p* < 0.05, n = 10)

For the second stage of growing experiments, this study assesses if kinetic growth parameters (μ_max_ and λ) were influenced by morel species. Wild morel isolates (FRE-5E, FRE-6F, FRE-I, FRE-L and FRE-M; Table 1) were cultivated in MEA-coconut (the culture media with the highest growth performance) and growth curves were fitted by primary modeling (Additional file 1: Table S1). Baranyi’s model growth kinetic values obtained for all the strains cultured in MEA-coconut (including *M. esculenta* CDBB-H-482 and isolate FRE-3C) were considered into the multivariate analysis of variance (MANOVA) and Duncan pos hoc analysis (α = 0.05). Wild isolate FRE-3C showed the higher lag phase (2.41 ± 0.04 days), followed by *M. esculenta* (2.1 ± 0.05 days). Isolates FRE-L, FRE-M, FRE-5E, FRE-6F showed similar values of λ (1.54 ± 0.24; 1.59 ± 0.28; 1.61 ± 0.01; 1.66 ± 0.02 days respectively, Fig. 5a).

Regarding growth rates estimations, isolate FRE-I exhibited the lowest values of μ_max_ (10.37 ± 0.58), whereas isolates FRE-L and FRE-M showed the highest values (33.53 ± 0.46; 33.40 ± 0.39; Fig. 5b; *p* < 0.05). Although isolate FRE-I did not have a long lag phase in comparison with isolate FRE-3C and *M. esculenta*, its growth rate was the slowest, which makes that this strain took the longest time to grow.

## Discussion

### Coconut water increase mycelial growth rate.

In the present study is proposed a novel culture media formulation for morels based on coconut water. According to the modeling outcomes, coconut water used as culture media supplement yields augmented mycelial growth rate of morels. Coconut water is a complex matrix, whose composition varies depending on the maturity stage, species variety and the site of cultivation. This complex mixture has been extensively used as a growth-promoting element on in vitro plant tissue cultures (Winarto and da Silva 2015; Prando et al. 2014; Prades et al. 2012; Peixe et al. 2007). Its use during mushroom cultivation is not so popular, but it has been successfully used for cultivating *Pleurotus djamor, P. salmoneostramineus, Ganoderma lucidum* and *Lentinus* sp*.* (Zurbano et al. 2017; Kalaw et al. 2016; Jacob et al. 2015; Magday et al. 2014). Although the use of coconut water has not been fully contemplated for mushroom cultivation, it is considered as an important element during domestication of wild fungi (Kalaw et al. 2016; Magday et al. 2014), now including morels.

Among the components of coconut water, sugars, minerals, free amino acids and growth promoting factors are considered those that makes it a suitable growth medium for microorganisms, plants and mushrooms (Jacob et al. 2015; Prando et al. 2014; Prades et al. 2012). Regarding sugars, sucrose, sorbitol, glucose and fructose are present in larger proportions in coconut water. Minor carbohydrates are galactose, xylose and mannose (Prades et al. 2012; Yong et al. 2009). Notice that sugar proportion varies depending on the maturity stages of coconut. The coconut water used in this study (6 or 7 month old) has a sugar content that varies from 2.7 to 7 g per 100 mL of water (Prades et al. 2012). Such variations may affect the performance of a coconut-based culture media or the reproducibility of a specific response such as fungal growth. During morel cultivation, the proportion of sugars is an important factor to consider. There are reports demonstrating that a combination of sugars not always leads to increasing growth rate (Winder 2006). Sugars like glucose and lactose reduce growth rate when combined with other sugars. Otherwise, culture broths containing sucrose result into an enhancement of growth velocity (Winder 2006). Although the wide variety of sugars and nutrients present in coconut water, when coconut water was combined with lactose or malt extract, an enhancement of growth was observed. Thus, the use of the combined MEA-coconut culture media enhances growth rate and results in a production of abundant vigorous mycelium.

Regarding mineral content, potassium is present in a major proportion in coconut water. In a lesser amount are calcium, magnesium, iron and zinc (Tan et al. 2014; Prades et al. 2012). Dulay et al. (2020) evaluated the effect of mineral salts on mycelial growth of three *Lentinus* species. Cultures supplemented with potassium and magnesium salts supported efficiently mycelial growth. Since mineral nutrition is an important factor to consider during morel mycelial growth (Liu et al. 2015), apparently coconut water is supplying required mineral for growth in the form of organic complexes (Alchoubassi et al. 2021) that may be easily taken up by morel mycelium.

Aside of sugars or minerals, phytohormones are one of the most important components of coconut water (Prades et al. 2012; Yong et al. 2009; Ge et al. 2005). Growth regulators in coconut water comprise cytokinins (zeatin-O-glucoside, dihydrozeatin-O-glucoside, kinetin, ZMP -trans-zeatin riboside 5’-monophosphate-), gibberellins (GA1 and GA3), auxins (IAA: indole-3-acetic acid) and abscisic acid (Prades et al. 2012; Yong, et al. 2009). The function of phytohormones has been extensively studied in plants, but their role on mushrooms growth is scarce even when some fungi produce phytohormones themselves (Vedenicheva et al. 2016; Barker and Tagu 2000; Dua and Jandaik 1979). In the case of cytokinins, remains unknown whether these hormones originated in fungi, plants or in both (Barker and Tagu 2000). In plants, cytokinins are involved in cell division, nutrient uptake, stimulation of the activity of shoots and meristems, seed germination, retard leaf senescence and they also participate in stress regulation (Li et al. 2020; Yong et al. 2009). Even cytokinins are produced endogenously in Fungi; research efforts have been only focused on studying their role during plant-pathogen interaction (Vedenicheva et al. 2016; Pozo et al. 2014; Walters and McRoberts 2006). Recently Vedenicheva et al. (2016) reported endogenous production of cytokinins and Dulay et al. (2020) reported the influence of phytohormones on *Lentinus* mycelial growth. The possibility that observed enhancement of morel mycelia growth could be a consequence of cytokinins or to other phytohormones in coconut water deserves further study. Additional investigations are needed to elucidate the functions of phytohormones on mushroom metabolism, including their role on growth and fructification.

### Coconut water promotes sclerotia formation.

Sclerotia formation during morel cultivation is highly sought but not often accomplished. Even though sclerotia formation process is still unrevealed; several efforts have been made to induce it in pure cultures (Guler and Ozkaya 2009; Winder 2006; Buscot 1993; Amir et al. 1992). Recently Liu et al. (2018) found that low concentrations of hydrogen peroxide increase sclerotia production, but high concentrations resulted in a low sclerotia production and the authors suggested that reactive oxygen species (ROS) play an important role in signaling more than creating cellular stress. Although composition of coconut endosperm is a complex matrix, its properties as inductor of cell division and cell differentiation are known (Prades et al. 2012; Yong et al. 2009). In this case, it is unknown which component of coconut water is promoting sclerotia production, but the use of the coconut endosperm may be an alternative to ensure their production during morel cultivation.

### Mycelial morphology

As expected, morel mycelial morphology varied accordingly with the culture media and it was described comparatively with respect to previously reported by Winder (2006). Among the different culture media, the morphology which is considered as typical for morel mycelia (He et al. 2015) was observed in MEA, MEA-lactose and MEA-coconut cultures. Baran and Boron (2017) characterized the morphology of *M. deliciosa* and *M. esculenta*, but the differences between both strains were barely visible. Herein, evident morphological differences among wild morel strains enrich the knowledge about the variation of morel mycelia depending on the culture media composition or isolates.

### Baranyi’s primary model is a valuable tool to assess mycelial growth

Although mathematical models were initially used to describe population growth, nowadays it is a useful tool to predict growth of pathogen moulds in food industry (Marín et al. 2008; Baty and Delignette-Muller 2004). Despite primary models provide valuable information, they are not commonly used to assess growth of edible mushrooms. Regarding morel characterization, growth is usually reported in terms of radial growth and kinetic parameters are not estimated.

Among the fitted models, linear model produces accurate predictions of growth, but it does not contemplate the lag phase which reduces the representativeness of the model for mycelium growth. Conversely, Baranyi’s model contemplates the lag phase because it has an adjustment function that delays exponential growth, so this phase can be better described by this model (Baty and Delignette-Muller 2004). This study takes advantage of primary models that can be a useful tool to assess optimization of morel mycelial growth. By using this tool, it could be possible to select strains with the most convenient kinetic parameters for the next stages of cultivation.

### Variation of kinetic growth parameters according to the morel species

Herein, interspecific differences on kinetic growth parameters (λ and µ_max_) were shown for the first time on morels. According to Buchanan and Cygnarowicz (1990), lag phase comprises the period were growth slows down. During this period, diverse physiological processes took place with the aim of providing an adaptation to the surrounding growing environment. Valenzuela-Cobos et al. (2017) fitted growth of *P. ostreatus, P. djamo*r and *Lentinula edodes* by using the Gompertz model and a nonlinear model. The values of λ ranged from 0.41 to 2.74 days. Such values are similar to those obtained in the present study (1.54 ± 0.24−2.41 ± 0.04 days), so the adaptation period is similar among morels and the *Basidiomycetes* above mentioned.

Valenzuela-Cobos et al. (2017) reported µ_max_ values for *P. ostreatus, P. djamo*r and *L. edodes* in a range from 0.44 to 1.27 days^−1^ for the modified Gompertz model and 0.26 to 1.69 day^−1^ by using the nonlinear model*.* The values of µ_max_ obtained for morels are one order of magnitude superior (10.37 ± 0.58 to 40 ± 0.39 mm d^−1^; Fig. 5b) to those reported by Valenzuela-Cobos et al. (2017). Notice that morel mycelium is considered a fast growing mycelium. Despite its rapid growth, morel mycelium is very susceptible and stops growing when there is a lack of nutrients or a competitor (Pilz et al. 2007). As well, in nature morel mycelium seems to grow better and fruit in disturbed areas (Loizides 2017; Loizides et al. 2015; Pilz et al. 2004). For these reasons it could be considered as a pioneer species of secondary succession. Pioneers of secondary succession are described as fast growth species that first colonize disturbed nutrient rich habitats where competition is reduced (Dalling 2008). The faster growing attribute of morels can be of great benefit during its artificial cultivation, but its susceptibility to competitors should be considered.

The results presented in this study demonstrate that using coconut water during morel cultivation and domestication may be an encouraging cheap alternative to produce dense good quality mycelium and promote sclerotia formation. The growing effect of coconut water was characterized not only by observational subjective analysis but using the variations of parameters corresponding to three different nonlinear models. A robust parametric modeling strategy allows the effective characterization of morels growth influenced by the presence of coconut water in the culture broth. A feasible explanation regarding which components of the water could be responsible of growth improvement is also given in this study. Even coconut is a complex matrix and it was not possible to determine which components promoted the observed growth enhancement and sclerotia formation, the results obtained in this study highlight the need for further investigations regarding to the influence of phytohormones (like cytokinins) on growth metabolism and fructification of morel mycelia.

## Supplementary Information

**Additional file 1:** **Table S1.** Growth rates (μ_max_, mm d-1) and lag phases (λ, days) of morel wild strains cultivated in different culture media according to linear model, Baranyi’s model and Logistic model (n=10).

## Data Availability

All data generated or analyzed during this study are available from the corresponding author on reasonable request.
