# Supplementary material for: A novel coconut-malt extract medium increases growth rate of morels in pure culture
Source: AMB Express. 2021 Dec 15;11:167. doi: 10.1186/s13568-021-01325-2 (PMC8674397; doi:10.1186/s13568-021-01325-2)
Supplement: Supplementary file 1 — Additional file 1: Table S1. Growth rates (μmax, mm d-1) and lag phases (λ, days) of morel wild strains cultivated in different culture media according to linear model, Baranyi’s model and Logistic model (n=10). [file 13568_2021_1325_MOESM1_ESM.pdf]

AMB Express

A novel coconut-malt extract medium increases growth rate of morels in pure culture

Fabiola R. Evangelista[a], Isaac Chairez[b], Sigfrido Sierra[c],
Hermilo Leal Lara[d], César Ramiro Martínez González[e], María Eugenia Garín Aguilar[f],
Gustavo Valencia del Toro[a*].

*Corresponding author. gvovaltor@gmail.com, tel.:+52 5557 296000 Ext. 56439.

[a]Laboratorio de Cultivos Celulares de la Sección de Estudios de Posgrado e Investigación, UPIBI, Instituto Politécnico Nacional, Barrio la Laguna s/n Ticomán, CP 07340 Mexico City, México.

[b]Unidad Profesional Interdisciplinaria de Biotecnología, Instituto Politécnico Nacional, Barrio la Laguna s/n Ticomán, CP 07340 Mexico City, México.

[c] Laboratorio de Taxonomía de Hongos Tremeloides (Heterobasidiomycetes), Departamento de Biología Comparada, Facultad de Ciencias, Universidad Nacional Autónoma de México, Ciudad Universitaria, Av. Universidad 3000, Coyoacán, 04510, Mexico City, Mexico.

[d]Departamento de Alimentos y Biotecnología, Facultad de Química, Universidad Nacional Autónoma de México, Cd. Universitaria, 04510 Mexico City, México.

[e]Posgrado en Biotecnología Agrícola, Instituto de Horticultura, Departamento de Fitotecnia, Universidad Autónoma de Chapingo, Chapingo, México, 56230, Texcoco, Mexico State, Mexico.

[f]Laboratorio de Farmacobiología, FES Iztacala, Universidad Nacional Autónoma de México, Av. de los Barrios No.1. Los Reyes Iztacala, CP 54090 Tlalnepantla, Mexico State, México.

Table S1 Growth rates (μ_{max}, mm d^{-1}) and lag phases (λ, days) of morel wild strains cultivated in different culture media according to linear model, Baranyi's model and Logistic model (n=10)

Strain	Culture media	Linear			Baranyi			Logistic			
		μ	λ	r^2	μ	λ	r^2	μ	λ	A	r^2
M. esculenta CDBB-H-482	Agar	13.45±.26	0.72±0.1	0.998±0.001	14.93±0.25	1.69±0.03	0.998±0.001	17.21±0.39	1.4±0.03	95.45±1.76	0.999±0.0002
	Agar-lactose	13.85±0.31	0.75±0.01	0.98±0.001	15.40±0.34	1.70±0.20	0.999±0.0004	13.85±0.31	1.37±0.01	100.58±2.49	0.999±0.0001
	MEA	15.21±0.21	0.93±0.01	0.97±0.002	18.03±0.33	1.99±0.04	0.995±0.001	20.96±0.39	1.82±0.02	114.73±3.18	0.999±0.0002
	MEA-lactose	15.18±0.17	0.95±0.01	0.96±0.002	18.15±0.38	2.04±0.05	0.99±0.001	20.88±0.31	1.85±0.02	120.48±3.17	0.999±0.0001
	Agar-coconut	14.89±0.31	0.86±0.20	0.94±0.005	19.86±0.29	2.42±0.05	0.997±0.0004	21.79±0.45	2.13±0.04	125.73±2.86	0.998±0.0004
	MEA-coconut	16.55±0.14	0.89±0.004	0.95±0.002	22.57±0.35	2.1±0.05	0.99±0.001	22.93±0.17	1.7±0.01	143.69±2.86	0.999±0.0001
	Agar-lactose-coconut	15.86±0.12	1.02±0.01	0.96±0.002	20.62±0.23	2.28±0.02	0.996±0.0004	22.94±0.25	2.01±0.02	117.63±4.11	0.998±0.0001
FRE-3C	Agar	9.42±0.46	0.88±0.05	0.93±0.01	12.97±0.69	2.79±0.11	0.98 ±0.003	13.54±0.65	2.3±0.07	212.15±59.19	0.99±0.002
	Agar-lactose	10.23±0.55	0.90±0.03	0.94±0.009	13.42±0.67	2.60±0.10	0.99±0.003	14.41±0.80	2.19±0.08	206.9±70.53	0.997±0.001
	MEA	14.67±0.31	1.07±0.01	0.94±0.01	19.70±0.32	2.44±0.04	0.996±0.002	21.61±0.43	2.16±0.03	139.21±20.15	0.997±0.0003
	MEA-lactose	13.53±0.41	0.99±0.02	0.94±0.007	18.03±0.49	2.39±0.98	0.99±0.001	19.47±0.56	2.08±0.07	145.04±20.05	0.997±0.0003
	Agar-coconut	14.70±0.29	1.03±0.01	0.95±0.004	19.31±0.35	2.35±0.06	0.997±0.001	21.29±0.47	2.08±0.38	117.78±4.87	0.997±0.0003
	MEA-coconut	15.64±0.25	1.08±0.01	0.95±0.004	21.13±0.43	2.41±0.04	0.997±0.0002	23.48±0.49	2.16±0.03	115.01±0.07	0.997±0.0004
	Agar-lactose-coconut	15.33±0.34	1.07±0.02	0.94±0.007	20.71±0.39	2.40±0.08	0.9961±0.001	22.91±0.47	2.15±0.06	129.36±9.49	0.998±0.0003
FRE-5E	MEA-coconut	18.93±0.39	0.83±0.02	0.98±0.002	21.66±0.51	1.61±0.01	0.999±0.0002	25.93±0.73	1.50±0.02	105.49±2.12	0.999±0.0001
FRE-6F	MEA-coconut	18.57±0.41	0.84±0.01	0.97±0.003	21.42±0.45	1.66±0.02	0.996±0.001	25.09±0.68	1.53±0.02	121.58±3.68	0.999±0.0001
FRE-I	MEA-coconut	9.55±0.51	0.48±0.06	0.96±0.005	10.37±0.58	1.70±0.03	0.97±0.005	11.87±0.64	1.45±0.06	423.94±148.6	0.995±0.001
FRE-L	MEA-coconut	26.31±0.05	0.84±0.01	0.98±0.003	33.53±0.46	1.54±0.24	0.9998±0.00003	38.58±0.30	1.42±0.03	103.04±2.15	0.999±0.0002
FRE-M	MEA-coconut	26.12±0.12	0.86±0.01	0.97±0.007	33.40±0.39	1.59±0.28	0.997±0.003	38.44±0.21	1.47±0.04	349.99±245.67	0.999±0.0001
